# Meis1 Is Required for the Maintenance of Postnatal Thymic Epithelial Cells

**DOI:** 10.1371/journal.pone.0089885

**Published:** 2014-03-04

**Authors:** Takehiro Hirayama, Yusuke Asano, Hajime Iida, Takeshi Watanabe, Takuro Nakamura, Ryo Goitsuka

**Affiliations:** 1 Division of Development and Aging, Research Institute for Biomedical Sciences, Tokyo University of Science, Chiba, Japan; 2 Center for Innovation in Immunoregulative Technology and Therapeutics, Kyoto University Graduate School of Medicine, Kyoto, Japan; 3 Department of Carcinogenesis, The Cancer Institute, Japanese Foundation for Cancer Research, Tokyo, Japan; Center for Molecular Biotechnology, Italy

## Abstract

Most epithelial tissues retain stem/progenitor cells to maintain homeostasis of the adult tissues; however, the existence of a thymic epithelial cell (TEC) progenitor capable of maintaining homeostasis of the postnatal thymus remains unclear. Here, we show that a cell population expressing high levels of Meis1, a homeodomain transcription factor, is enriched in TECs with an immature cellular phenotype. These TECs selectively express genes involved in embryonic thymic organogenesis and epithelial stem cell maintenance, and also have the potential to proliferate and differentiate into mature TEC populations. Furthermore, postnatal inactivation of *Meis1* in TECs caused disorganization of the thymic architecture, which ultimately leads to premature disappearance of the thymus. There was an age-associated reduction in the proportion of the TEC population expressing high levels of Meis1, which may also be related to thymic involution. These findings indicate that Meis1 is potentially involved in the maintenance of postnatal TECs with progenitor activity that is required for homeostasis of the postnatal thymus.

## Introduction

The thymus is a primary lymphoid organ populated by several distinct epithelial and mesodermal cell lineages, including two thymic epithelial cell (TEC) subsets, cortical keratin 8 (K8)-positive TEC (cTEC) and medullary keratin 5 (K5)-positive TEC (mTEC), bone marrow-derived dendritic cells and macrophages, and endothelial cells. All of these cell types are required for proper intrathymic T cell proliferation, differentiation and selection [1], [2]. The epithelial component of the thymus is formed from the endoderm of the third pharyngeal pouch, which buds out into the underlying mesenchyme, a process that begins on day 10.5 of mouse embryonic development (E10.5). Epithelial cells initially cluster to form the thymic primordium, and then perithymic mesenchymal cells and lymphoid precursors migrate into this rudiment, initiating normal thymic development. Recently it has been demonstrated that embryonic TEC stem/progenitors arise as cells with cTEC markers, and then acquire mTEC markers [3]–[5]. TEC stem/progenitor cells have been formally identified by their capacity to differentiate into the full range of epithelial cells characteristic of a functional thymic microenvironment. Thus, a single K5^+^ K8^+^ epithelial cell from an E12 embryonic thymus can differentiate into both mTECs and cTECs when injected into a normal fetal thymic lobe [6]. Several transcription factors, including FoxN1 [7], [8], Hoxa3 [9], Pax1 [10], Pax9 [11], [12], Eya1 [13], Pbx1 [14] and p63 [15], [16], are important for thymic epithelial cell proliferation and differentiation, and altered expression or activity of any of them leads to severe defects in thymic organogenesis and development. Among them, p63 is of particular interest because this factor, especially one of its isoforms, ΔNp63, has been demonstrated to be selectively expressed in TEC stem/progenitor cells and required for their proliferation potential [15], [16].

In the postnatal thymus, the cortical and medullary architecture of the TEC microenvironment displays dynamic alterations in response to various physiological and pathological stimuli. The existence of TEC stem/progenitor cells that function to maintain the homeostasis of the postnatal thymic microenvironment has long been a matter of debate [17], [18]. Most epithelial tissues, including skin and intestine, that retain potential stem/progenitor cells are functionally maintained throughout the lifetime of the organism, whereas atrophy in the epithelial compartment of the thymus begins around puberty and progresses with age until, eventually, the functional microenvironment of this organ has been completely degraded in the aged organism [19]. However, like other epithelial tissues, the postnatal thymus appears to preserve an intrinsic ability to regenerate itself by responding to external stimuli, such as castration and cytokine administration [20], [21], suggesting the existence of stem/progenitor cells capable of maintaining homeostasis of the postnatal thymus. This idea is supported by the observation that postnatal reactivation of *FoxN1* in single keratin 14 (K14)^+^ cells of *FoxN1*-deficient thymic cysts led to the development of small but functional thymic tissues with normally organized architecture [22]. In this regard, it has recently been demonstrated that Cbx4, a member of the Polycomb group family, is a critical regulator for both embryonic and postnatal TEC proliferation and functions [23], some of which are regulated by its interaction with p63, suggesting a possible link between embryonic and postnatal TEC stem/progenitor cells.

During a microarray search for transcription factors responsible for TEC regeneration, *Meis1* (myeloid ecotropic viral integration site 1) was identified as one of the genes up-regulated in regenerating TECs following transient thymic atrophy induced by irradiation or dexamethasone treatment [24]. *Meis1* encodes a homeodomain transcription factor of the TALE (three-amino-acid-loop extension) subfamily, and is homologous to the *Drosophila* Homothorax gene [25]. By interacting with Pbx, a member of another TALE homeodomain subfamily, Meis becomes intimately associated with certain Hox transcription factors and modulates the specificity and affinity of their DNA binding in several Hox-dependent developmental programs [26], including vertebrate hindbrain development and limb morphogenesis [27], [28]. Meis1 has also been reported to maintain the undifferentiated state and expansion of retinal progenitor cells [29], [30], olfactory epithelial cells [31] as well as embryonic hematopoietic stem cells [32], [33]. With respect to TEC differentiation, it is noteworthy that Hoxa3 and Pbx1, both of which are required for embryonic thymic organogenesis [9], [14], are functional and physical partners of Meis1.

In the present study, we used *Meis1-EGFP* reporter mice and tamoxifen-inducible epithelial-specific *Meis1*-conditional knockout mice to analyze the phenotype, function and molecular signature of *Meis1*-expressing TECs. We show here that postnatal thymic medulla contains two TEC populations with distinct Meis1 expression levels. The Meis1^high^ TEC population represents immature TECs, as defined by cellular phenotypes, the expression patterns of genes involved in embryonic thymic organogenesis and epithelial stem cell maintenance, and progenitor-progeny analyses. Furthermore, Meis1 is required for maintenance of the postnatal thymus because its absence caused premature thymic disappearance and the proportion of the Meis1^high^ TEC population decreases with age. Taken together, these findings demonstrate potential involvement of Meis1 in maintenance of the progenitor activity of postnatal TECs required for homeostasis of the postnatal thymus.

## Results

### Expression of Meis1 in the thymic microenvironment

**Figure 1:**
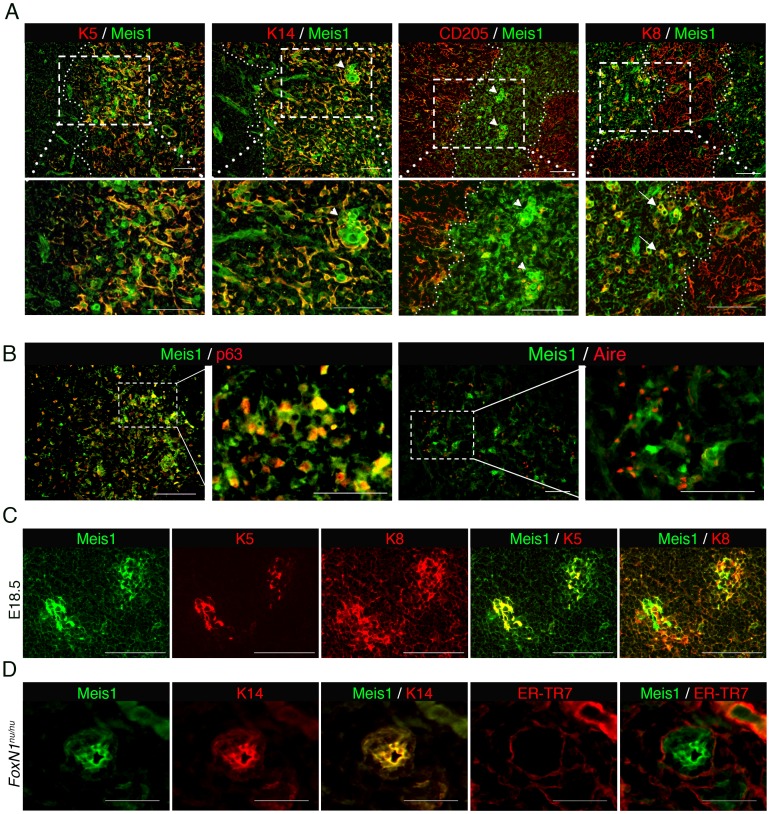
Expression of Meis1 in a small population of mTECs. (**A**–**B**) Immunohistochemical analysis to localize Meis1-expressing cells in the postnatal thymus. Serial tissue sections of the thymus from 4-week-old wild-type mice were stained with the Meis1-specific antibody, in combination with anti-K5, K8, K14 or CD205 antibodies (**A**) and with anti-CD11c, CD31, p63, ER-TR7 or Aire antibodies (**B**). Broken lines indicate the cortico-medullary border. (**C**) Triple-color immunostaining of E18.5 thymus using anti-Meis1, K5 and K8 antibodies. (**D**) Triple-color immunostaining of the adult thymic rudiment from *FoxN1* ^nu/nu^ mice using anti-Meis1, K14 and ER-TR7 antibodies. Images shown are representative of three independent experiments. Scale bars, 100 µm.

To understand the function of Meis1 in TEC differentiation, we first examined its expression in the thymus. In the postnatal thymus (at four weeks of age), *Meis1* transcripts were selectively detectable in EpCAM^+^ TECs, particularly in MHC II^+^ UEA-1^+^ mTECs, but not in thymocytes, including the CD4^+^ or CD8^+^ single-positive (SP), CD4^+^CD8^+^ double-positive (DP) and CD4^−^CD8^−^ double-negative (DN) subpopulations (**Figure S1**). Immunohistochemical analysis using an anti-Meis1 antibody revealed that Meis1^+^ cells were predominantly localized in the medulla and were very rare in the cortex, as defined by co-localized staining with K5 and K14, markers for mTECs, and by K8 and CD205, cTEC markers (**Figure 1A**). There were at least two distinguishable Meis1^+^ TECs in the medulla: round and relatively small cells expressing relatively high levels of Meis1 (Meis1^high^) and stellate cells with lower Meis1 expression (Meis1^low^) (**Figure 1A** **, indicated by arrowheads**). Meis1^low^ cells, forming the majority of Meis1^+^ cells in the medulla, were positive for K5 and K14, whereas some Meis1^high^ cells were positive for K8, which is expressed in rare mTECs (**Figure 1A** **, indicated by arrows**). Furthermore, p63, a transcription factor known to mark TEC stem/progenitor cells [15], [16], and Aire, a transcription factor expressed in mature mTECs [34], were differentially expressed in Meis1^high^ and Meis1^low^ cells, respectively (**Figure 1B**). Although Meis1 expression was not detectable in CD11c^+^ thymic dendritic cells, CD31^+^ vascular endothelial cells as well as ER-TR7-positive fibroblasts in the postnatal thymus were found to express Meis1 (**Figure S2**). In the thymus at embryonic day 18.5 (E18.5), Meis1 was highly expressed in TECs positive for both K5 and K8 (**Figure 1C**). We also examined the thymic rudiment present in nude mice that have a loss-of-function mutation in the *FoxN1* gene. Their K14^+^ TECs, which have been shown to retain the capacity to regenerate complete thymic epithelial cell compartments [22], were found to express high levels of Meis1 (**Figure 1D**). These findings demonstrate that Meis1 is expressed at distinct levels in two populations of postnatal mTECs, and suggest that mTECs expressing high levels of Meis1 might include immature mTECs or TEC progenitors.

### Epithelial-specific deletion of Meis1 causes thymic atrophy

**Figure 2:**
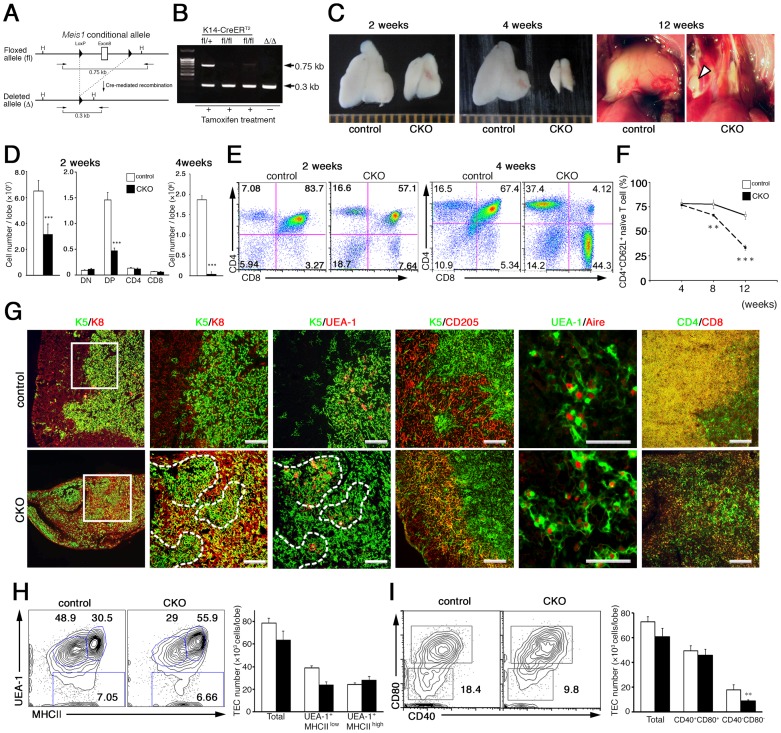
Epithelial-specific deletion of *Meis1* causes thymic atrophy. (**A**) A diagram depicting the floxed *Meis1* allele. *LoxP* sites (arrowheads) were inserted into intronic sites flanking exon 8 of the *Meis1* gene. PCR primers for verifying the Cre-mediated deletion of the *loxP*-flanked fragment are indicated by arrows. (**B**) Confirmation of *Meis1* deletion in K14-CreER^T2^ mice. DNAs from sorted EpCAM^+^ CD205^−^ TECs from K14-CreER^T2^ *Meis1* ^fl/fl^ and *Meis1* ^fl/+^ mice that were either treated (+) or untreated (−) with tamoxifen were subjected to PCR analysis using primer pairs shown in ***A***. DNAs from *Meis1* ^Δ/Δ^ mice were used as controls. (**C**) Gross appearance of thymus lobes from CKO and control mice 2 weeks (left) and 4 weeks (middle) post induction of *Meis1* deletion. A macroscopic view of a mediastinum of CKO and control mice 12 weeks post induction of *Meis1* deletion is also shown (right). The thymus lobes of control mice were positioned above the heart, whereas in the CKO the thymic lobes had completely disappeared. White arrowheads indicate ectopic lymph nodes. (**D**) Total cell numbers of total thymocytes (left) and subpopulations of thymocytes at different developmental stages (middle) from CKO (black bars) and control (white bars) mice 2 weeks post induction of *Meis1* deletion. Numbers of total thymocytes (right) from CKO (black bars) and control (white bars) mice 4 weeks post induction of *Meis1* deletion are also shown. Data are the mean and standard deviation of three independent experiments. ^***^, p<0.001. (**E**) Representative FACS profiles of thymocyte differentiation in CKO and control mice 2 weeks (left two panels) and 4 weeks (right two panels) post induction of *Meis1* deletion. (**F**) The proportion of CD44^−^ CD62L^+^ naïve CD4 T cells among total CD4 T cells in the peripheral blood of CKO (dotted line) and control (solid line) mice. Data are the mean and standard deviations of three independent experiments. ^**^, p<0.01 and ^***^, p<0.001. (**G**) Immunohistochemical analysis of the thymus from CKO and control mice 4 weeks post induction of *Meis1* deletion. Tissue sections were double stained with the indicated antibody combinations. Broken lines indicate cortico-medullary borders. Data are representative of 3 independent experiments. Scale bars, 100 µm. (**H**–**I**) Representative FACS profiles of TECs from K14-CreER^T2^ and control mice 4 days after tamoxifen treatment. Numbers indicate percentage of cells within the indicate gates. Bars show the cell number of the indicated EpCAM^+^ TECs from CKO (black) and control (white) mice. Shown are the mean and standard deviation from three independent experiments. ^**^, p<0.01.

As the early embryonic lethality of germline deletion of the *Meis1* gene precludes any study of postnatal maintenance of the thymus [32], [33], we generated mice harboring conditional alleles of *Meis1* (*Meis1^fl^*), in which exon 8 of the *Meis1* gene encoding the Meis1 homeodomain is flanked by *lox*P sites (**Figure 2A**). Given the high expression of *Meis1* in immature mTECs, we chose to study the consequence of Meis1 ablation in these cells by crossing the *Meis1^fl^* conditional-knockout strain with the *K14* gene promoter-driven tamoxifen-responsive K14-CreER^T2^ transgenic line, which effects highly efficient excision of *lox*P-flanked DNA in immature epithelial cells after induction with tamoxifen or 4-hydroxytamoxifen (4-OHT) [35]. Three intraperitoneal injections of tamoxifen into K14-CreER^T2^ *Meis1^fl/fl^* mice were sufficient to induce complete deletion of the floxed *Meis1* locus in EpCAM^+^ CD205^−^ TECs (**Figure 2B**).

We analyzed the thymic architecture of adult K14-CreER^T2^ *Meis1^fl/fl^* mice at 2, 4 and 12 weeks after the tamoxifen treatment (hereafter referred to as CKO) and compared it to that of similarly treated K14-CreER^T2^ *Meis1^fl/+^* or *Meis1^fl/fl^* littermates (hereafter referred to as controls). Two weeks after the induction of *Meis1* deletion, the size and total cell number of the thymus in the Meis1-CKO mice were about half of those in the controls (**Figures 2C and D**). The phenotype became more severe by four weeks post-induction (PI); Meis1-CKO mice harbored only a small thymic remnant and the total cell number was less than 1% of the control thymus (**Figures 2C and D**). Eventually, the thymus of the CKO mice completely disappeared by 12 weeks PI (**Figure 2C** **, right panels**). Flow cytometric analysis revealed significant and selective reduction of the proportion and the absolute number of CD4^+^ CD8^+^ DP T cells in CKO mice at two weeks PI (**Figures 2D and E**). To understand the selective reduction in DP T cells upon Meis1 loss, we examined early T-cell progenitors and their differentiation through the DN stage. There were no significant alterations in the proportions and cell numbers of these immature T cells, including early T-cell progenitors (**Fig. S3**), suggesting that the arrival of bone marrow-derived early T-cell progenitors to the thymus as well as early T-cell development until the DN4 stage in the thymus is not affected by Meis1 loss in postnatal TECs. Furthermore, all the thymocyte populations were significantly diminished in the CKO at four weeks PI (**Figure 2D**), and the reduction in the proportion of CD4^+^ CD8^+^ DP T cells became more severe than was observed at two weeks (**Figure 2E**). The proportion of CD62L^+^ CD44^−^ naïve CD4^+^ T cells in peripheral blood also gradually declined from 4 to 12 weeks PI (**Figure 2F**), indirectly implicating the defective function of the thymic microenvironment in supporting T-cell development.

Immunohistochemical analyses revealed that the architecture of the medulla and cortex was severely disorganized in the CKO mice after four weeks PI (**Figure 2G**). The K5^+^ medullary compartment in the CKO thymus was severely reduced in size, although it still retained UEA-1^+^ and Aire^+^ mature mTECs, suggesting that the mature mTEC compartment was not severely affected by Meis1 loss (**Figure 2G**). Furthermore, the K5^+^ K8^+^ as well as K5^+^ CD205^+^ TECs, but not K8^+^ TECs that are distributed in the cortex of control thymus, occupied the cortex compartment surrounding the medullary region of the CKO thymus (**Figure 2G**). Although the molecular mechanisms by which Meis1 loss in the K14^+^ mTEC compartment causes cTEC alterations remain unclear, the phenotypic alterations observed in the cTEC compartment might be accompanied by profound reductions in the cell number of CD4^+^ CD8^+^ DP cells that preferentially reside in the cortical compartment of the thymus. The appearance of K5^+^K8^+^ cells in the cortex was detectable at least at 2 weeks after *Meis1* deletion, at which time point the level of CD4^+^ CD8^+^ DP cell proportion was already lower than that of control DP cell population (**Figure S4**). At 12 weeks PI, we occasionally observed two small thymus-like remnant tissues bilaterally in the thoracic cavity where the normal thymus had been located prior to the induction of *Meis1* deletion (**Figure 2C**, **arrowheads**). However, these structures turned out to be ectopic lymph nodes (LN) that contained no K5^+^ or K8^+^ epithelial cells but instead displayed the typical LN accumulation of B220^+^ B cells and CD3^+^ T cells (**Figure S5**).

To identify the initial target cell populations affected by *Meis1* loss, we examined cellular changes in the TEC population by flow cytometry at the early phase of *Meis1* deletion. Four days after the induction of *Meis1* deletion, the numbers of EpCAM^+^ TECs in the CKO were comparable to those in control mice. However, the proportion and absolute number of immature mTECs (MHCII^low^ UEA-1^+^ and CD40^−^ CD80^−^) were profoundly reduced in the CKO mice. By contrast, *Meis1* deletion only marginally affected the number of mature mTECs (MHCII^high^ UEA-1^+^ and CD40^+^ CD80^+^) as well as MHCII^dull^ UEA-1^−^ cTECs (**Figures 2H and I**), suggesting the possibility that the initial loss of ^-^immature mTECs induced by *Meis1* deletion results in disorganization of the thymic microenvironment. Taken together, these findings demonstrate that Meis1 is functionally required for maintenance of the postnatal thymic microenvironment.

### High levels of Meis1 expression mark immature TEC population in the postnatal thymus

**Figure 3:**
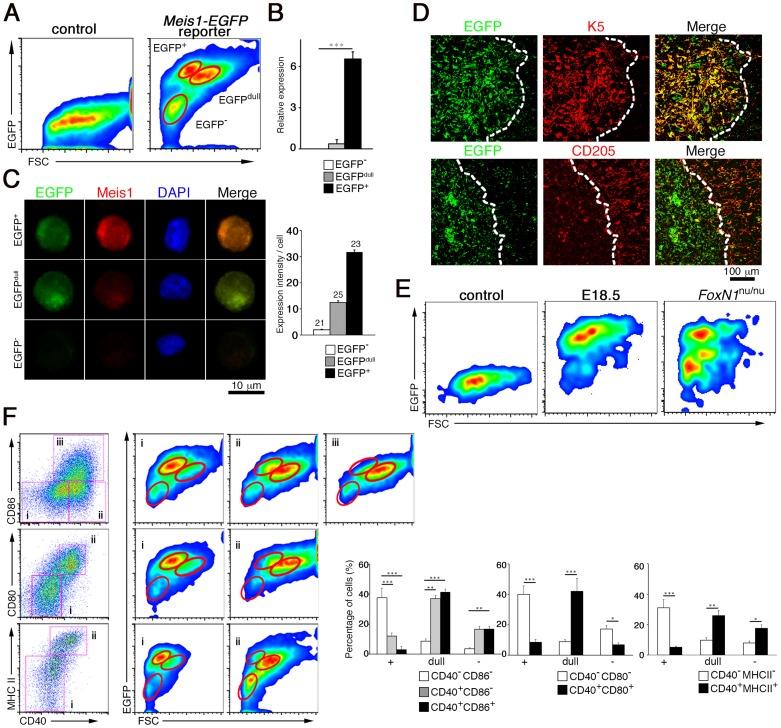
The Meis1^high^ TEC population represents immature TECs in the postnatal thymus. (**A**) Representative FACS profiles showing EGFP expression in TECs from 4-week-old *Meis1-EGFP* reporter mice. EpCAM^+^ CD45^−^ TECs were analyzed for EGFP fluorescence in combination with the forward scatter (FSC) profile. TECs from wild-type littermates were used as controls for background EGFP fluorescence. The TEC populations with distinct EGFP fluorescence intensities (EGFP^+^, EGFP^dull^ and EGFP^−^) are marked by red circles. (**B**) The level of Meis1 mRNA transcripts in EGFP^+^, EGFP^dull^ and EGFP^−^ TECs. Data were normalized to *ß-actin* and the level of Meis1 transcript in the whole thymus was arbitrarily set to 1. Shown are the mean and standard deviations of three independent samples. (**C**) Representative image of sorted EGFP^+^, EGFP^dull^ and EGFP^−^ TECs stained with anti-EGFP (green) and anti-Meis1 (red) antibodies. Nuclei were counterstained with DAPI. Bar graphs represent signal intensities of Meis1 in sorted single EGFP^+^, EGFP^dull^ and EGFP^−^ TEC. Data were normalized to DAPI intensities and the level of each signal in EGFP^−^ cells was arbitrarily set to 1. Numbers above the bars indicate the number of cells examined. (**D**) Immunohistochemical analysis of EGFP expression in the thymus from 4-week-old *Meis1-EGFP* reporter mice. Tissue sections were double stained with the indicated antibody combinations. Broken lines indicate cortico-medullary borders. (**E**) FACS profiles showing EGFP fluorescence in TECs from *Meis1-EGFP* reporter mice at E18.5 (middle) and on the *FoxN1* ^nu/nu^ background (right). (**F**) FACS profiles showing the expression of EGFP with differentiation markers in TECs from 4-week-old *Meis1-EGFP* reporter mice. Cells were gated on CD45^−^ EpCAM^+^ TECs and divided into developmental stages using the combination of cell surface markers CD40 with CD86 (upper panels), CD40 with CD80 (middle panels) or CD40 with MHC II (bottom panels). EGFP fluorescence in each gate is shown along with forward scatter profiles. Bar graphs shown on the right are the proportion of the indicated marker-positive cells among EGFP^+^, EGFP^dull^ and EGFP^−^ TECs. Data are the mean and standard deviation of three independent experiments.

To understand how Meis1 functions to maintain the postnatal thymic microenvironment, we characterized TECs expressing *Meis1* at the single cell level using *Meis1-EGFP* reporter mice [36]. These mice carry a bacterial artificial chromosome (BAC) transgene (RP23-306E8) corresponding to ∼80 kb upstream and ∼30 kb downstream of the mouse *Meis1* gene, into which an *EGFP* cDNA was introduced just 5′ of the *Meis1* translation start site, thus destroying any transgenic expression of *Meis1* from the BAC. *Meis1* expression was thus detectable in single cells by using EGFP fluorescence as a surrogate marker. Consistent with our RT-PCR results (**Figure S1**), EGFP expression was observed in EpCAM^+^ TECs but not in thymocytes (**Figure S6**). A more detailed analysis using a combination of EGFP fluorescence and forward scatter (FSC) showed that EpCAM^+^ TECs can be divided into three populations with different EGFP expression levels and cell sizes: EGFP^+^ FSC^low^, EGFP^dull^ FSC^high^ and EGFP^−^ FSC^low^ (**Figure 3A**). When we compared endogenous Meis1 expression with EGFP fluorescence in these cell populations, *Meis1* mRNA transcripts were significantly detectable only in the EGFP^+^ FSC^low^ cells (**Figure 3B**), and the level of Meis1 protein expression, estimated from staining intensities, was approximately threefold higher in EGFP^+^ FSC^low^ cells than in EGFP^dull^ FSC^high^ cells (**Figure 3C**). These findings clearly indicate that EGFP^+^ FSC^low^ cells are the TECs that have gone on to transcribe the *Meis1* gene to generate high levels of Meis1 protein, while EGFP^dull^ FSC^high^ cells appear to be EGFP^+^ FSC^low^ descendants because they contain a substantial amount of Meis1 protein but have its gene transcription shut off. Immunohistochemical analyses also confirmed that the EGFP reporter was mainly expressed in the thymic medulla (**Figure 3D**). Furthermore, similar to endogenous Meis1 expression in the embryonic and *FoxN1* ^nu/nu^ thymus (**Figures 1C and D**), both EGFP^+^ FSC^low^ and EGFP^−^ FSC^low^ TECs were detectable in the thymus at E18.5 and in the thymic cysts of adult *Meis1-EGFP* reporter mice on the *FoxN1* ^nu/nu^ background (**Figure 3E**). Thus, it is highly likely that EGFP^+^ FSC^low^, EGFP^dull^ FSC^high^ and EGFP^−^ FSC^low^ cells correspond to endogenous Meis1^high^, Meis1^low^ and Meis1^−^ TECs observed by immunohistochemistry, respectively (**Figure 1A**; for simplicity hereafter referred to as EGFP^+^, EGFP^dull^ and EGFP^−^).

To take advantage of the *Meis1-EGFP* reporter mice for characterization of Meis1-expressing cells, we further examined the phenotype of TECs with distinct Meis1 expression levels. As shown in **Figure 3F**, the majority of cells among the most immature TECs (CD40^−^ CD86^−^) were EGFP^+^, whereas EGFP^dull^ cells predominated in intermediate (CD40^+^ CD86^−^) and mature (CD40^+^ CD86^+^) TECs. A similar pattern of EGFP fluorescence was observed when we subdivided the TEC populations based on CD40 and CD80 or MHCII expression; CD40^−^ CD80^−^ and CD40^−^ MHCII^−^ immature TECs were enriched for the EGFP^+^ cells, while CD40^+^ CD80^+^ and CD40^+^ MHCII^+^ mature TECs were enriched for the EGFP^dull^ cells (**Figure 3F**). The EGFP^−^ cells did not show clear distribution patterns in terms of the TEC maturation stages. These results suggest that the EGFP^+^ cell population that selectively transcribes the *Meis1* gene in the postnatal thymus corresponds to immature TECs that are the most sensitive to *Meis1* deletion.

### Gene expression patterns in TEC populations expressing different levels of Meis1

**Figure 4:**
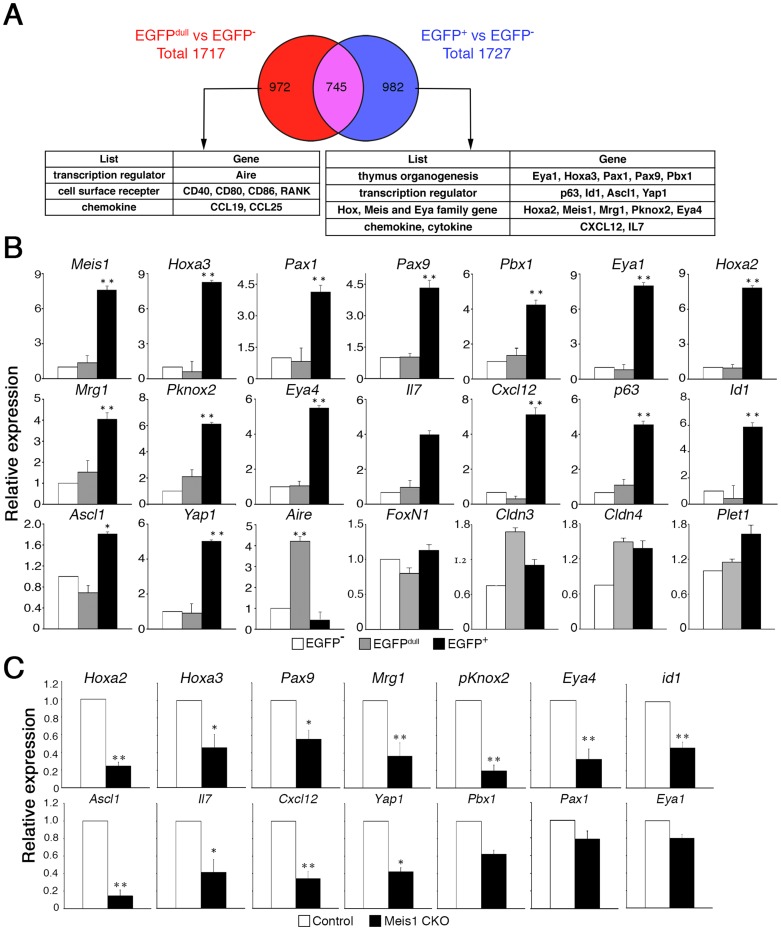
Expression profile of genes differentially enriched in Meis1^low^ and Meis1^high^ TECs. (**A**) A Venn diagram showing the number of transcripts (gene probes) of up-regulated thymic stromal signature genes shared and distinct between EGFP^+^ and EGFP^dull^ TECs sorted from the thymus of 4-week-old *Meis1-EGFP* reporter mice. The number of transcripts within each subpopulation is shown inside the Venn diagram. The full gene lists are shown in Table S1 and S2. Representative genes differentially expressed in EGFP^+^ and EGFP^dull^ TECs relative to EGFP^−^ TECs are also shown. (**B**) Gene expression levels between EGFP^+^, EGFP^dull^ and EGFP^−^ TECs were validated by quantitative RT-PCR. Histograms show the indicated transcripts in sorted TECs of EGFP^+^ (black bars), EGFP^dull^ (gray bars) and EGFP^−^ (white bars). Data were normalized to *ß-actin* expression and the level of each transcript in EGFP^−^ cells was arbitrarily set to 1. Shown are the mean and standard deviation of at least three independent experiments. *p<0.05, and **p<0.01. (**C**) Alterations of gene expression in TECs induced by *Meis1* loss were analyzed by quantitative RT-PCR. Histograms show the indicated transcripts in sorted TECs from K14-CreER^T2^ *Meis1* ^fl/fl^ mice (white bars) and control mice (black bars) 4 days post tamoxifen treatment. Data were normalized to *ß-actin* expression and the level of each transcript in TECs from control mice was arbitrarily set to 1. Data are the mean and standard deviations of at least 3 independent experiments. *p<0.05 and **p<0.01.

To obtain a better understanding of the nature of the differences in the TEC populations with distinct Meis1 expression levels, we performed comparative gene expression profiling by microarray analysis, and compared these data with the previously reported expression profiles of thymus stromal signature genes [37]. To define the thymus stromal signature genes differentially expressed in EGFP^+^ and EGFP^dull^ TECs, we compared the genes differentially expressed 1.7-fold or more between EGFP^+^ and EGFP^−^ TECs with the genes differentially expressed between EGFP^dull^ and EGFP^−^ TECs (**Figure 4A**; see **Tables S1** and **S2** for the full gene lists). We also validated the differential expression of a subset of these genes by quantitative PCR (**Figure 4B**). In addition to *Meis1* itself, the genes differentially up-regulated in EGFP^+^ TECs included those involved in embryonic thymus organogenesis [9]–[14], such as *Hoxa3*, *Pax1*, *Pax9*, *Pbx1* and *Eya1*, and in epithelial stem cell maintenance and differentiation [16], [38]–[40], such as *p63*, *Id1*, *Ascl1* and *Yap1* (**Figures 4A and B**). It is also worth noting that other *Hox*, *Meis* and *Eya* family genes (*Hoxa2*, *Mrg1*, *Pknox2* and *Eya4*), previously unrecognized as potentially important in the development of TECs, were also up-regulated in EGFP^+^ cells. Furthermore, genes affecting TEC functions [41], [42], *IL-7* and *Cxcl12*, were also up-regulated in EGFP^+^ cells. By contrast, the genes up-regulated in the EGFP^dull^ TECs included *Aire*, a gene required for promiscuous gene expression in relatively mature mTECs [34], and genes encoding cell surface molecules expressed on mature TECs, such as CD40, CD80, CD86 and RANK (*Tnfsf11a*), a finding consistent with the mature phenotype of EGFP^dull^ TECs (**Figure 3F**). Together, these findings demonstrated that EGFP^+^ TECs are a unique postnatal TEC population highly expressing key transcription factors essential for embryonic TEC differentiation and proliferation and also for the maintenance of epithelial stem/progenitor cells.

To obtain further insight into the genetic effect of *Meis1* loss on the EGFP^+^ TEC population, we examined alterations in the gene expression profiles of TECs from Meis1-CKO and controls at four days after tamoxifen treatment, and compared the genes that were down-regulated 1.7-fold or more by *Meis1* deletion in TECs with the gene set differentially expressed in EGFP^+^ TECs (**Table S3**). Expression of some of the genes identified by microarray was also verified by quantitative PCR analysis (**Figure 4C**). Among genes up-regulated in EGFP^+^ TECs, most of the genes encoding transcription factors/co-activators, including *Hoxa2*, *Hoxa3*, *Pax9*, *Mrg1*, *pKnox2*, *Eya4*, *Id1* and *Ascl1*, were down-regulated upon *Meis1* deletion, although *Pax1*, *Pbx1* and *Eya1* expression levels were not significantly altered (**Figure 4C**). In addition, genes encoding IL-7 and Cxcl12 were significantly reduced in the Meis1-CKO TECs. These findings demonstrate that Meis1 is one of the components of transcription factor networks that are essential for the maintenance of postnatal thymic microenvironments that support T-cell differentiation.

### Differentiation potentials of the Meis1^high^ TEC population

**Figure 5:**
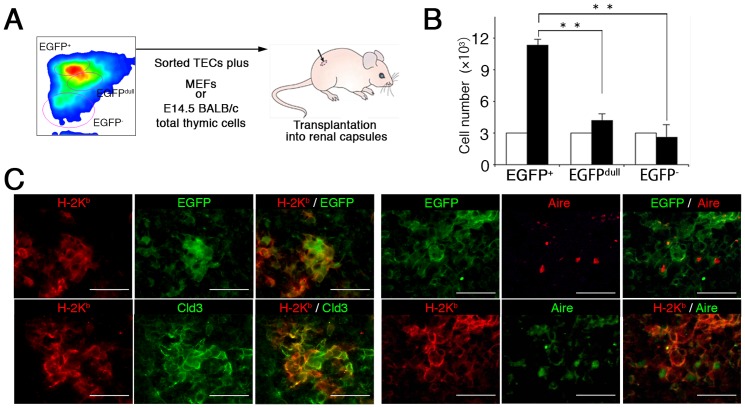
The Meis1^high^ TEC population retains the progenitor activity. (**A**) Experimental strategy for analysis of the progenitor potential of postnatal TECs with different *Meis1* expression levels. TECs with distinct Meis1 expression levels (EGFP^+^, EGFP^dull^ and EGFP^−^) were sorted from the thymus of 4-week-old *Meis1-EGFP* reporter mice; 3×10^3^ cells from each fraction were mixed with 1×10^5^ MEFs and absorbed into the scaffold. The resultant cell-embedded scaffold was implanted into the renal capsules of nude mice, and 4 weeks later the implanted scaffolds were analyzed. (**B**) Total numbers of CD45^−^ EpCAM^+^ TECs from the renal implants. The input (open bars) and recovered (filled bars) cell numbers are shown. Data are the mean and standard deviation of three independent experiments. ^**^, p<0.01. (**C**) Immunostaining of the reagregated E14.5 BALB/c thymus containing EGFP^high^ TECs with the indicated antibody combinations. Scale bars, 100 µm.

We further examined differentiation potentials of the Meis1^high^ TEC population by using a cell transfer model that has been successfully applied for the generation of artificial lymph nodes [43]. TECs with distinct EGFP expression levels were sorted from adult *Meis1-EGFP* reporter mice (four weeks of age), and were incorporated into biocompatible cubic sponge-like collagenous scaffolds, which were then transplanted under the renal capsule of recipient mice (**Figure 5A**). Four weeks after the transplantation, the total number of EpCAM^+^ cells recovered from the grafted scaffold containing EGFP^+^ TECs increased about fourfold, whereas no cell expansion was observed in grafted scaffolds containing EGFP^dull^ or EGFP^−^ TECs (**Figure 5B**). Since the EGFP^+^ TECs themselves failed to generate the fully developed thymus-like epithelial organization with the medulla and cortex compartments in this transfer model (data not shown), we examined the differentiation capacity of postnatal EGFP^+^ TECs (H-2K^b^) by incorporating them into an *in vitro* reaggregated E14.5 BALB/c (H-2K^d^) thymus, which was transplanted under the renal capsule of recipient mice. Immunohistochemical analyses revealed that EGFP^+^ TEC-derived H-2K^b^ cells were detected in the thymic medulla as Claudin3^+^ as well as mature Aire^+^ TECs (**Figure 5C**), whereas the grafts embedded with EGFP^dull^ cells or EGFP^−^ cells contained no H-2K^b^ cells (data not shown), suggesting the differentiation potentials of postnatal immature TECs expressing high levels of Meis1 to mature TECs.

### Age-related aleration in the Meis1^high^ TEC population

**Figure 6:**
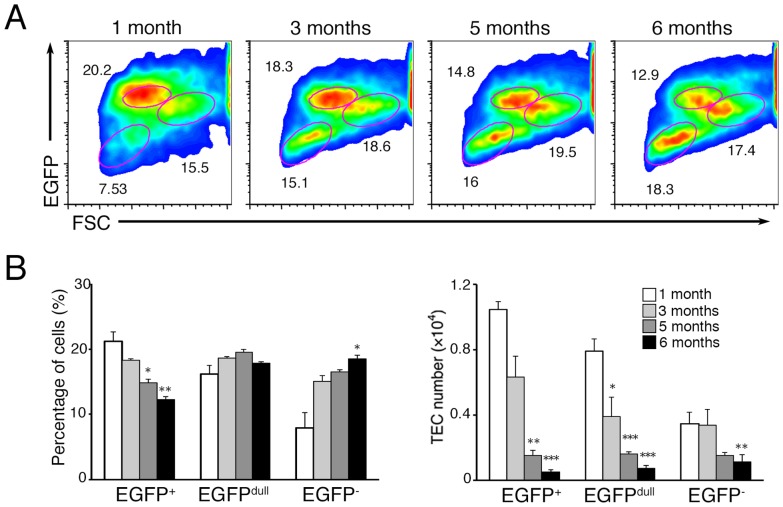
Age-associated reduction in the Meis1^high^ TECs. (**A**) Representative FACS profiles showing the expression of *Meis1* by CD45^−^ EpCAM^+^ TECs with age. TECs from mice of the indicated ages were analyzed for expression of EGFP by flow cytometry. Data are representative of three independent experiments. (**B**) The percentage and total cell numbers of EGFP^+^, EGFP^dull^ and EGFP^−^ TECs in mice of different ages; 1 month (white bars), 3 months (light gray bars), 5 months (dark gray bars) and 6 months (black bars). Bars represent the mean and standard deviations of the percentage (left) and cell numbers (right) of each TEC population from four independent experiments. Significance relative to 1-month-old mice; ^***^, p<0.001; ^**^, p<0.01; ^*^, p<0.05.

Given that the thymic architecture dynamically changes with age, we finally addressed the physiological relevance of Meis1 function in age-associated alterations in the thymic microenvironment. There was a gradual decrease in the proportion of EGFP^+^ cells beginning at ∼3 months of age and the reduction of these immature EGFP^+^ TECs was clearly apparent at 5 and 6 months of age (**Figures 6A and B**), indicating an age-associated reduction in an immature Meis1^high^ TEC population in the postnatal thymus. This reduction in immature Meis1^high^ TECs may result in age-associated exhaustion of progenitor cells capable of maintaining homeostasis of the postnatal thymus.

## Discussion

In the present study, we demonstrate that expression of Meis1 in TECs is required for maintenance of the postnatal thymic microenvironment, as the postnatal loss of *Meis1* in the K14^+^ TEC compartment causes not only a reduction in the size of the medulla but also formation of a disorganized cortex, eventually leading to complete disappearance of the thymus.

Our analyses of endogenous Meis1 and reporter gene expression in the thymus have shown that Meis1 is expressed in subpopulations of mTECs, and this heterogeneity in Meis1 expression levels marks distinct mTEC populations with respect to their differentiation and maturation stages. The Meis1^high^ TEC population (EGFP^+^), corresponding to the cells going on to transcribe the *Meis1* gene, represents immature TECs (CD40^−^ MHCII^−^ CD80^−^ and CD86^−^) with up-regulated expression of genes encoding transcription factors/co-activators that are essential for embryonic thymus organogenesis, such as *Hoxa3*, *Pax1*, *Pax9*, *Pbx1* and *Eya1*. In contrast, the Meis1^low^ TEC population (EGFP^dull^), corresponding to the cells in which *Meis1* gene transcription has already been shut down, represents mature TECs with a CD40^+^ MHCII^+^ CD80^+^ CD86^+^ phenotype and high expression of *Aire*, a finding that is consistent with a previous observation that TECs expressing Aire are postmitotic terminally differentiated mTECs [44]. Furthermore, Meis1^high^ TECs were found to proliferate and to differentiate into mature TECs when ectopically transplanted under the renal capsule of nude mice. These findings suggest that the Meis1^high^ TEC population in the postnatal thymus may include a postnatal TEC progenitor. In this regard, it has recently been demonstrated that postnatal TEC clones cultured under particular conditions *in vitro* and displaying a medullary phenotype (K5^+^, K14^+^) with enriched expression of p63 have the capacity to reconstitute a thymic microenvironment under the kidney capsule of nude mice [45]. Furthermore, these cultured TEC clones were shown to exhibit high expression of key transcription factors/co-activators involved in thymus organogenesis, such as Eya1, Six1, Pax1, Pax9 and Hoxa3, most of which are also enriched in the Meis1^high^ TEC population described here. Thus, observed similarities in gene expression profiles of the Meis1^high^ TEC population with the reported cultured TEC clones with stem cell activity further reinforce the idea that the Meis1^high^ TEC population contains postnatal TEC progenitors.

With regard to the function of Meis1 in postnatal TEC progenitor activity, p63 is of particular interest among the transcription factors potentially regulating TEC progenitors because it is preferentially expressed in TEC stem/progenitor cells to regulate their potential to self-renew [16]. Although p63 is enriched in the Meis1^high^ immature TEC population (Figures 1B and 4B) and expression of both Meis1 and p63 is independent of the activity of FoxN1, p63-deficient thymic phenotypes were apparently different from those in Meis1 deficiency. The loss of p63 in TECs selectively caused a proliferation defect in TECs, but did not disturb TEC differentiation, as both K5^+^ mTECs and K8^+^ cTECs were normally distributed in the p63-deficient thymus [15], [16]. Furthermore, p63-deficient mice displayed severe reductions in the absolute numbers of thymocytes without a disturbance in the relative proportion of CD4^+^ CD8^+^ DP as well as CD4^+^ and CD8^+^ SP thymocytes. Thus, p63 appears to exhibit selective control of the proliferation potentials of TEC stem/progenitors, but not their maturation and functions supporting T-cell differentiation in the thymus. In sharp contrast, postnatal Meis1 loss in TECs caused not only maturation defects in thymocyte differentiation, particularly at the CD4^+^ CD8^+^ DP stage, but also the disorganization of TEC compartments, which is characterized by the early loss of immature TECs and also by the appearance of K5^+^K8^+^CD205^+^ atypical TECs in the cortex. In this regard, one intriguing TEC phenotype that may link the phenotypic difference between Meis1 and p63 deficiency is that caused by deficiency in Cbx4, a Polycomb protein that directly interacts and potentially functions in conjunction with p63 [23]. The Cbx4-deficient mice displayed the hypoplastic, but morphologically intact, thymic microenvironment in the perinatal period, which highly resembles that observed in p63 deficiency [23]. However, similar to the Meis1-deficient phenotypes describe in the present report, loss of Cbx4 in the TEC compartment resulted in the appearance of K5^+^K8^+^ double-positive TECs in the postnatal thymus, which was also accompanied by the preferential reduction of CD4^+^CD8^+^ DP thymocytes, although such phenotypic alterations in TECs as well as in thymocyte development were not observed in the neonatal and embryonic thymus of the Cbx4 deficient mice [23]. Although it remains to be clarified whether Cbx4 functions in conjunction with p63 in the postnatal thymus, Meis1, like Cbx4, may have roles in the maintenance of the TEC functions supporting T-cell differentiation in the postnatal thymus. Further investigation of the functional interplay between Meis1 and p63/Cbx4 is clearly warranted to resolve this issue.

Aging causes thymic involution, but the genetic pathways that control this process are poorly understood. It has been reported that TECs from aged mice had reduced proliferative capacity and a higher rate of apoptosis [46]. Postnatal TEC division predominantly occurs in the mTEC population [47], and reduction in the proportion of dividing TECs with age causes the changes in stromal composition that characterize the involuted thymus. In this regard, we observed that the proportion of the immature Meis1^high^ TEC population is reduced with age. This age-associated reduction in immature Meis1^high^ cells potentially containing postnatal TEC progenitors seems to be one of the potential mechanisms involved in age-associated thymic involution. In this regard, p63, in association with FoxN1, has also recently been revealed to participate in age-related thymic involution [48]. Further studies on the function of Meis1 in TECs, in relation to the p63-FoxN1 regulatory axis, could provide an avenue for clarifying the molecular mechanism underlying age-associated thymic atrophy.

In conclusion, we provide evidence that, in the postnatal thymus, the high expression of Meis1 marks an immature TEC population with potential progenitor activities, and that Meis1 is required for homeostatic maintenance of the postnatal thymus. Further dissection of the function of Meis1 in the transcription factor networks regulating maintenance of the postnatal thymic microenvironment, particularly in association with p63 and Cbx4, should shed light on the role of epithelial stem/progenitor cells in the homeostasis and regenerative capability of the postnatal thymus, and may lead to the development of novel therapeutic strategies to regenerate thymic functions in pathological conditions.

## Materials and Methods

### Ethics Statement

All animal experiments were carried out under the ethical guidance of Tokyo University of Science, and protocols were reviewed and approved by the Tokyo University of Science Animal Care and Use Committee.

### Mice

Details in the generation of mice carrying floxed allele of the *Meis1* gene (*Meis1^fl/fl^*) will be described elsewhere. In brief, the targeting vector containing a 0.9-kb genomic fragment immediately upstream of the *lox*P-flanked 0.5-kb fragment containing exon 8 of the *Meis1* gene and a 6.5-kb DNA fragment immediately downstream of the gene was electroporated into E14 ES cells, and drug-resistant colonies were screened for homologous recombination. Targeted clones were injected into C57BL/6 blastocysts and the resultant chimeric mice were bred to produce progeny having germ line transmission of the mutated allele. By crossing with *EIIa*-Cre transgenic mice [49], the *loxP*-flanked neomycin-resistant gene cassette was removed from the allele. The resultant mice were backcrossed at least 8 times onto the C57BL/6 background and then were crossed with K14-CreER^T2^ transgenic mice on a pure C57BL/6 background [35]. *EIIa*-Cre and K14-CreER^T2^ mice on a C57BL/6 background were obtained from The Jackson Laboratory and Dr. Pierre Chambon, respectively. *Meis1-EGFP* BAC-transgenic reporter mice, generated by the GENSAT BAC transgenic project [36], were obtained from the Mutant Mouse Regional Resource Center and were backcrossed at least 5 times onto the C57BL/6 background. BALB/c nude mice were purchased from CLEA Japan. To induce *Meis1* deletion, 3-week-old K14-CreER^T2^ *Meis1^fl/fl^* and K14-CreER^T2^ *Meis1^f//+^* mice were injected intraperitoneally on 3 consecutive days with 1 mg of tamoxifen (Final concentration 20 mg/ml, Sigma-Aldrich, St Louis, MO) that was prepared following the manufacturer's instructions by completely dissolving Tamoxifen into 200 µl of 100% ethanol at 55°C, adding 1800 µl of warmed sunflower oil, and mixing them well by vortexing. Primer sequences and PCR conditions for genotyping are available upon request.

### TEC preparation

Thymic fragments were stirred gently in RPMI-1640 (Wako Pure Chemical Industries) medium for 15 min at 4°C to remove free thymocytes and then transferred to fresh medium containing 1.25 mg/ml collagenase/dispase with 0.01 mg/ml DNase I (Roche Diagnostics, Basel, Switzerland), and incubated for 15 min at 37°C and subjected to vigorous pipetting. The last three steps were repeated twice, discarding the supernatant each time, and incubation was continued until tissue digestion was complete. Released cells were filtered to remove clumps. For enrichment of the TEC populations, an immunomagnetic separation technique was performed using anti-CD45 coated beads and anti-Ter119 coated beads (Miltenyi Biotec) to deplete hematopoietic cells and erythroblasts. Purified TECs were sorted with a FACSArea™II or FACSvantage (BD Bioscience, San Diego, CA).

### Flow cytometry analysis

Single cell suspensions from the indicated organ were stained with a combination of FITC-, PE-, -APC, -APC-Cy7 and biotin-conjugated antibodies, followed by streptavidin-PE, -APC, -PE-Cy7, -and PerCP-Cy5.5 (eBioscience, San Diego, CA). The conjugated and unconjugated antibodies specific to the following antigens were purchased from BD Biosciences and eBioscience: TCRσß (H57-597), CD3e (145-2C11), CD4 (L3T4), CD8a (53-6.72), B220 (RA3-6B2), CD62L (MEL-14), CD44 (IM-7), CD45 (104), CD40 (HM40-3), CD80 (16-10A1), CD86 (GL1), MHC II (M5/114.15.2), CD205 (DEC205), TER119, CD16/CD32 (2.4G2). A biotin-labeled anti-EpCAM (G8.8) antibody was kindly provided from Dr. H. Kawamoto. Thymocytes and TECs were blocked by anti-CD16/32 antibody before staining and incubated with the antibodies for 20 min at 4°C. Data were collected on a FACSCalibur or FACSCanto™II flow cytometers (BD Bioscience) and analyzed using FlowJo software (TreeStar, Ashland, OR). FACSArea™II or FACSvantage (BD Bioscience) was used for cell sorting.

### Immunofluorescence analysis

Frozen thymus lobes embedded in OCT compound (Sakura Finetek) were cut into 6 µm sections and stained with hematoxylin and eosin. For multicolor analysis, frozen sections were fixed 4% paraformaldehyde or 100% cold acetone. Sections were incubated with primary antibodies diluted in blocking buffer overnight at 4°C. The following primary antibodies were used: rabbit anti-Meis1 (Abcam), rabbit anti-keratin 5 (Covance Research, Berkeley, CA, USA), rabbit anti-keratin 14 (Covance), rabbit anti-EGFP (Molecular Probes, Invitrogen), mouse anti-keratin 8 (PROGEN), mouse anti-CD4 (eBioscience), mouse anti-CD8 (eBioscience), rat anti-CD31 (eBioscience), rat anti-ER-TR7 (Abcam), biotinylated UEA-1 (Vector Laboratories), biotinylated anti-mouse CD11c (eBioscience), goat anti-Aire (D-17; sc-17986, Santa Cruz, CA, USA), biotinylated anti-mouse CD205 (eBioscience) and rabbit anti-p63 (Santa Cruz Biotechnology). Secondary antibodies were Alexa Fluor 488 conjugated anti-rabbit antibody (Molecular Probes), Alexa Fluor 633-conjugated anti-mouse (Molecular Probes), Alexa Fluor 555 conjugated anti-rat (Molecular Probes) or Alexa Fluor 555 conjugated streptavidin (Molecular Probes). Anti-rabbit Keratin5 and Keratin14 antibodies were also directly labeled with Alexa Fluor 555 using a Zenon Antibody Labeling Kit according to the manufacturer's instructions (Molecular Probes). Nuclei were counterstained with Prolong Gold antifade reagent with DAPI (Molecular Probes). The FACS-sorted cells were spun onto glass slides with a cytospin centrifuge, and immunostained with anti-Meis1 and EGFP antibodies. For quantification of staining intensities, average intensities of the Meis1, EGFP and DAPI signals in the cells were measured with MetaMorph software (Molecular Device). The Meis1 and EGFP signals in the cells were normalized to the DAPI signal. Images were analyzed with a BIOREVO BZ-9000 microscope (KEYENCE).

### Transplantation

TECs were obtained from the thymus of 4-week-old *Meis1-EGFP* reporter mice by sorting on the basis of EGFP expression levels. Each of TEC populations (3×10^3^ cells) was mixed with MEFs (1×10^5^ cells) and then suspended in 10 µl of PBS. The cell suspension was then placed onto a piece of the collagen sponge (CS-35; KOKEN), which was squeezed several times to absorb cells into the scaffold. The matrix-embedded cells were maintained on ice and kept moist throughout this procedure and immediately implanted into the renal subcapsular spaces of nude mice. For thymic reaggregation, each of TEC populations (3×10^3^ cells) was reaggregated with total enzyme-digested cells from E14.5 BALB/c thymic lobe (3×10^5^ cells) *in vitro* on membrane filters (Whatman), cultured for 24 hours, and then transplanted into the renal subcapsular spaces of nude mice. Four weeks after transplantation, the grafts were collected for immunohistochemical and flow cytometric analyses.

### Microarray analysis

RNA was isolated using the Qiagen RNeasy micro kit (Qiagen) from TECs with distinct *Meis1* expression levels (EGFP^high^, EGFP^int^ and EGFP^−^) sorted from 4-week-old *Meis1-EGFP* reporter mice (pools of 6 mice; 2 pools per sample) or TECs from tamoxifen-treated K14-CreER *Meis1* ^fl/fl^ and sham-treated K14-CreER *Meis1* ^fl/+^ mice (pools of 6 mice; 2 pools per genotype). Ten ng of total RNA was amplified using the WT-Ovation™ Pico RNA Amplification system (NuGEN Technologies, Inc.) and labeled using the Genomic Enzymatic Labeling Kit (Aligent Technologies). Labeled probes were hybridized on 4×44 K Whole Mouse Genome Oligo Microarrays (Aligent) and scanned with an Aligent Microarray Scanner. Microarray signals and background information were retrieved using Feature Extraction Software (v.9.5.3.1). All data analyses were performed using the GeneSpring software GX11.0.2 (Aligent). Genes with a raw *P*-value<0.01 and a fold-change greater than 1.7-fold were defined as differentially expressed. Array data will be available at Gene Expression Omnibus (accession number; GSE30826).

### RT-PCR analysis

Total RNA from sorted TECs was prepared and reverse transcribed using SuperScript VILO cDNA Synthesis system (Invitrogen, Carlsbad, CA). The cDNA was PCR-amplified, electrophoresed, and visualized with ethidium bromide. For quantitative analysis, real-time RT-PCR was performed with EXPRESS SYBR GreenER (Invitrogen) and ABI 7500 Fast thermocycler (Applied Biosystems, Foster City, CA). Amplified signals were confirmed to be single bands over gel electrophoresis, and normalized to *ß-actin*. Primer sequences and PCR were listed in **Table S4**.

### Statistical analysis

Statistical significance was calculated with the unpaired two-tailed Student's *t*-test.

## Supporting Information

Figure S1 **RT-PCR analysis of the endogenous** ***Meis1*** **expression in the thymus.**
(TIF)

Figure S2 **Expression of Meis1 in thymic microenvironment other than TECs.**
(TIF)

Figure S3 **Flow cytometric analysis of CD4^−^ CD8^−^ double-negative thymocyte differentiation two weeks after** ***Meis1*** **deletion.**
(TIF)

Figure S4 **Immunohistochemical analysis of TECs two weeks after** ***Meis1*** **deletion.**
(TIF)

Figure S5 **Lymph node-like tissues ectopically developed upon Meis1 loss.** Immunohistochemistry (left panels) and representative FACS profiles (right panels) of lymphoid-like remnants in CKO mice and the thymus from control mice 12 weeks post induction of *Meis1* deletion. Tissue sections were double stained with the indicated antibody combinations. Scale bars, 100 µm.
(TIF)

Figure S6 **Flow cytometric analysis of Meis1 expression in the thymus by using** ***Meis1-EGFP*** **reporter mice.**
(TIF)

Table S1 **Thymic stromal signature genes differentially expressed in Meis1^high^ TECs compared with Meis1^−^ TECs.**
(XLS)

Table S2 **Thymic stromal signature genes differentially expressed in Meis1^low^ TECs compared with Meis1^−^ TECs.**
(XLS)

Table S3 **Thymic stromal signature genes preferentially expressed in Meis1^high^ TECs that were down-regulated upon** ***Meis1*** **deletion.**
(XLS)

Table S4 **List of PCR primer sequences.**
(XLS)
