# Supplementary figures and images for: Meis1 Is Required for the Maintenance of Postnatal Thymic Epithelial Cells
Source: PLoS One. 2014 Mar 4;9(3):e89885. doi: 10.1371/journal.pone.0089885 (PMC3942356; doi:10.1371/journal.pone.0089885)

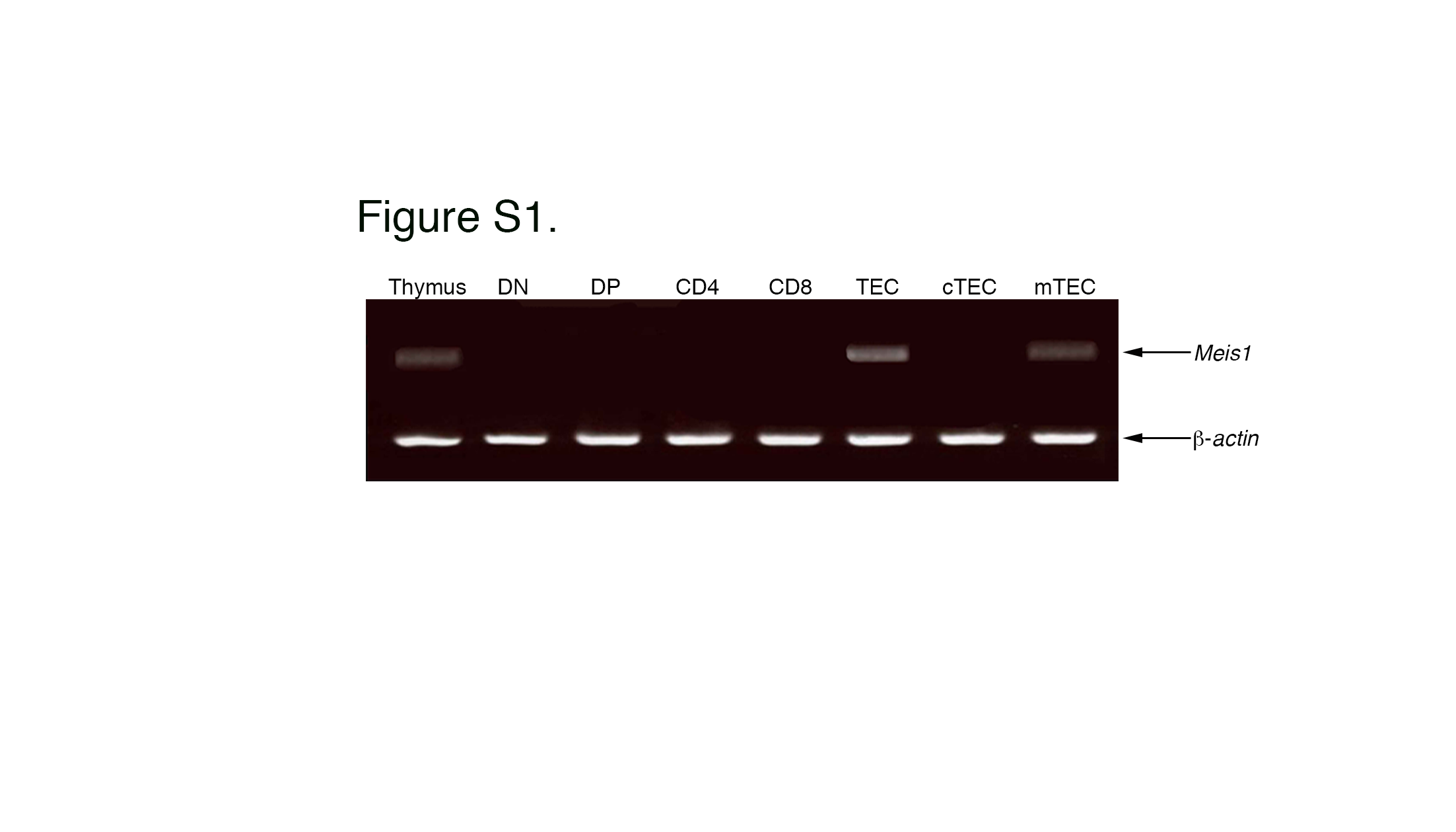

Supplement: Figure S1 — RT-PCR analysis of the endogenous Meis1 expression in the thymus. (TIF) [file pone.0089885.s001.tif]

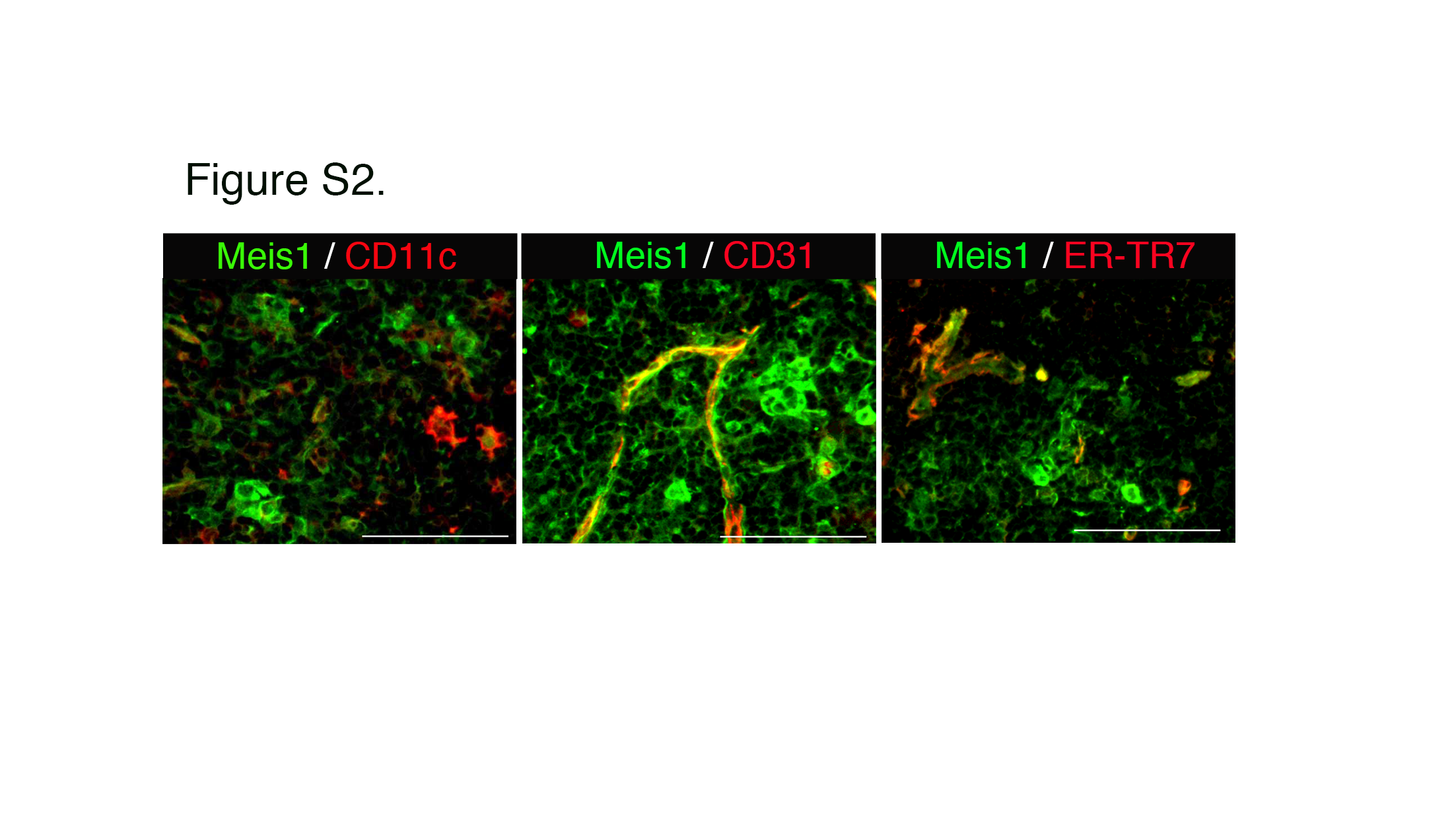

Supplement: Figure S2 — Expression of Meis1 in thymic microenvironment other than TECs. (TIF) [file pone.0089885.s002.tif]

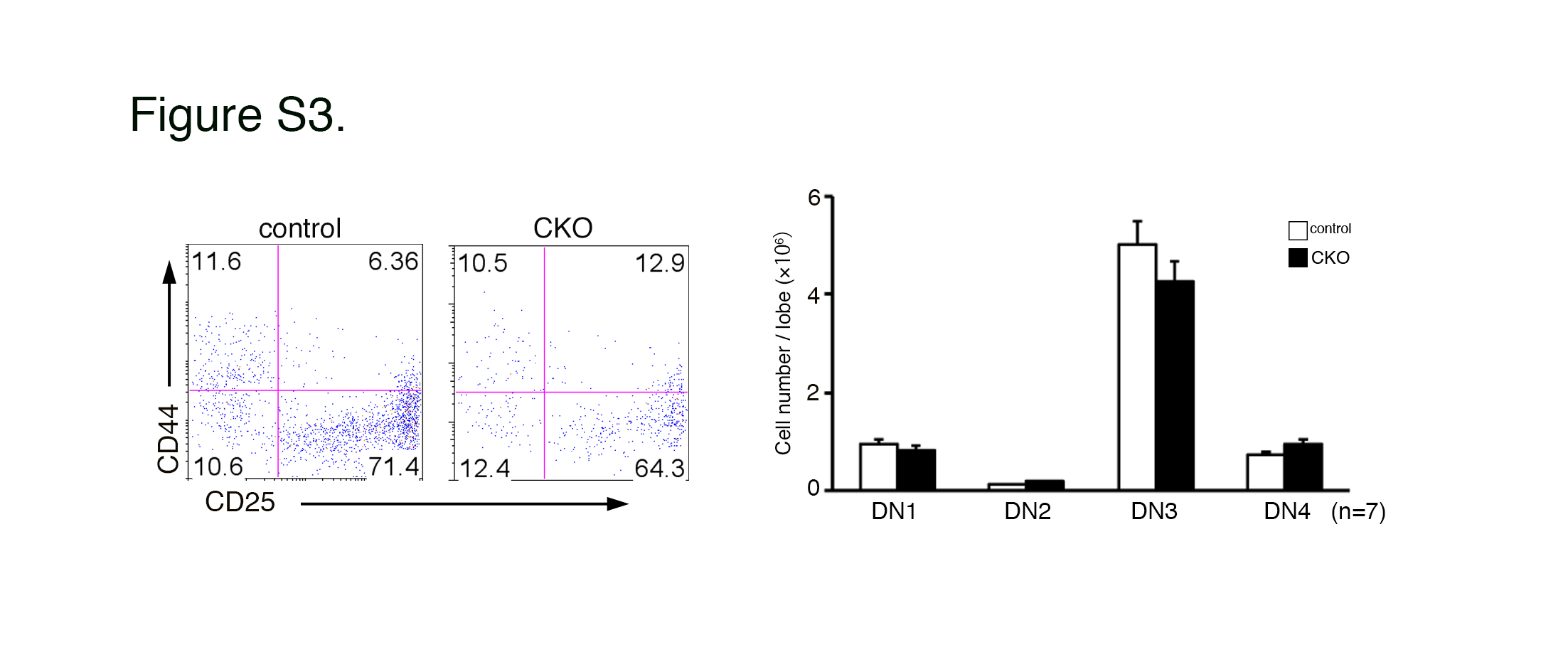

Supplement: Figure S3 — Flow cytometric analysis of CD4− CD8− double-negative thymocyte differentiation two weeks after Meis1 deletion. (TIF) [file pone.0089885.s003.tif]

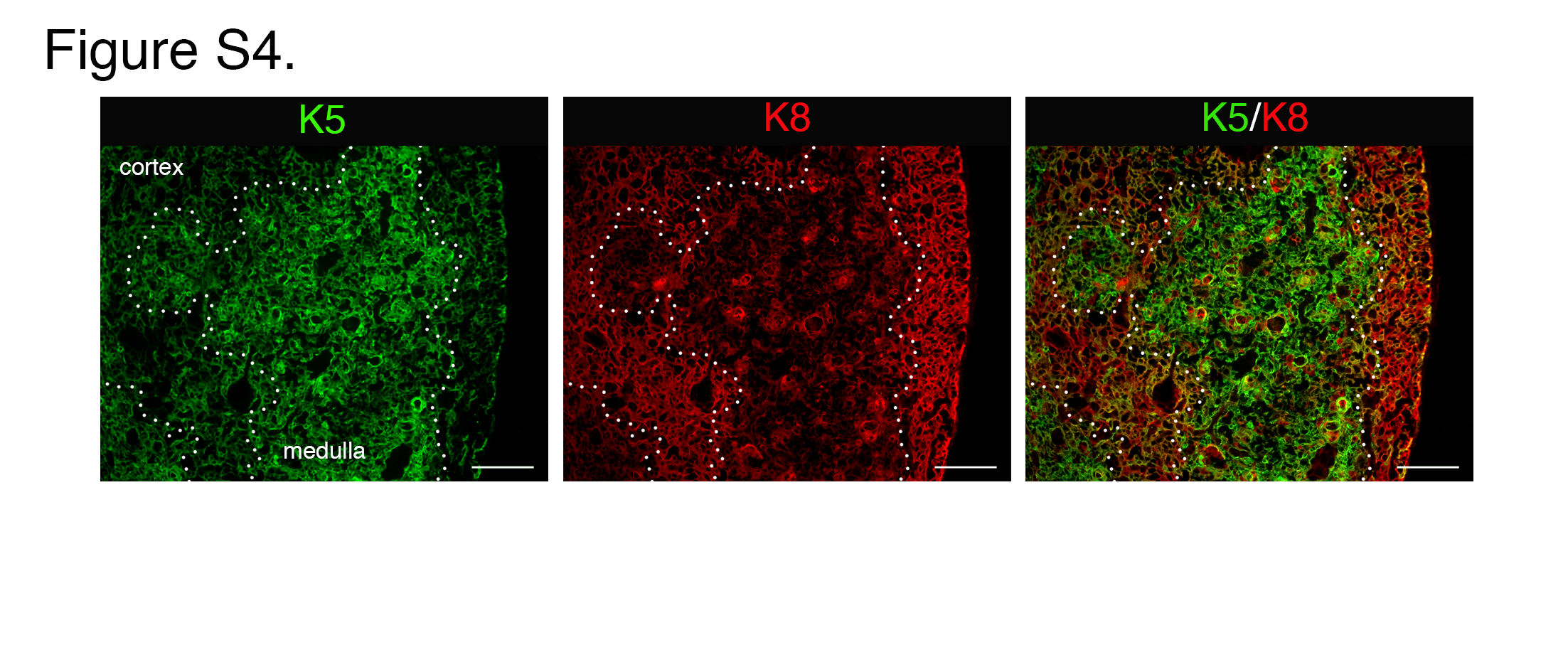

Supplement: Figure S4 — Immunohistochemical analysis of TECs two weeks after Meis1 deletion. (TIF) [file pone.0089885.s004.tif]

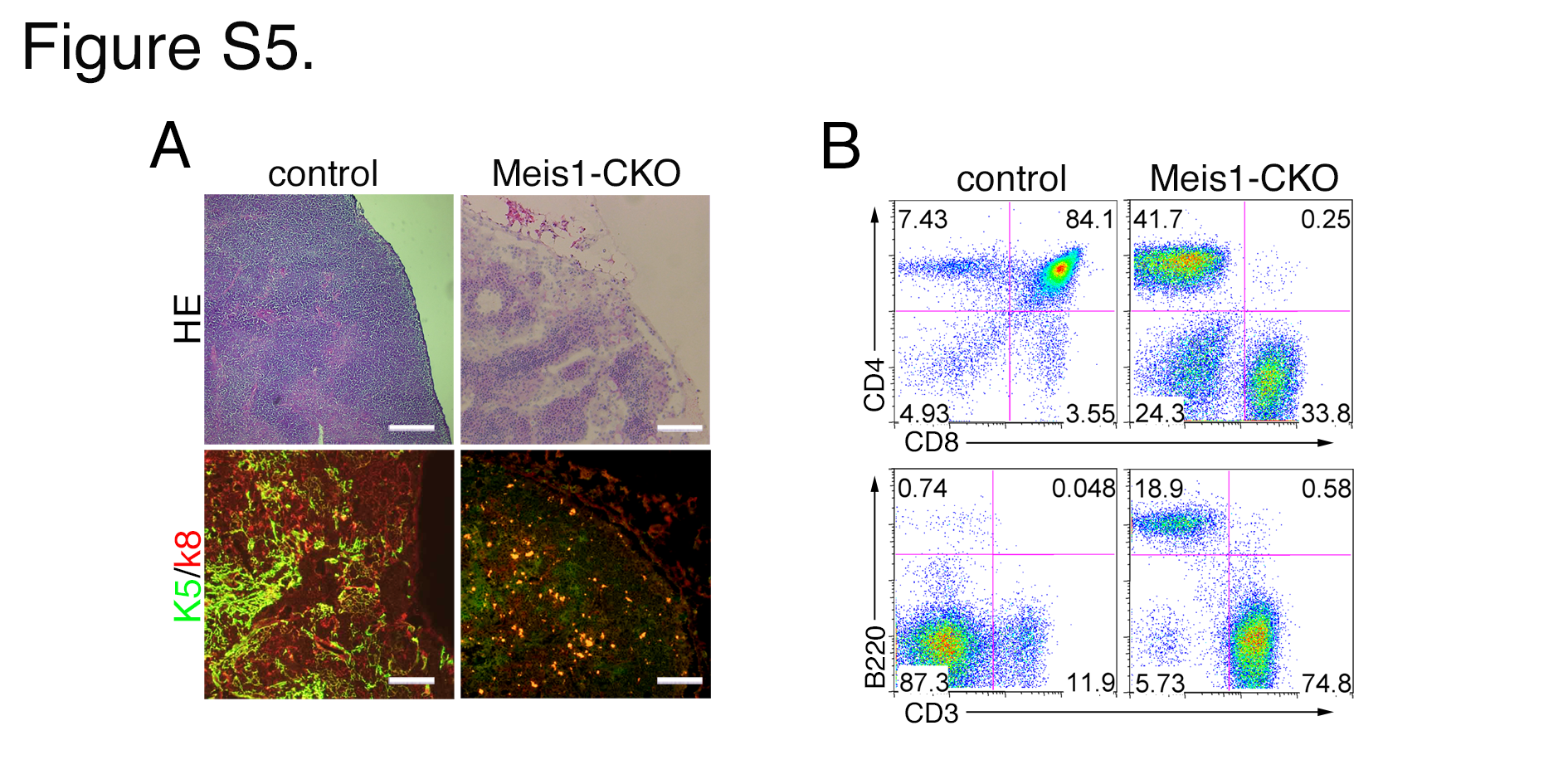

Supplement: Figure S5 — Lymph node-like tissues ectopically developed upon Meis1 loss. Immunohistochemistry (left panels) and representative FACS profiles (right panels) of lymphoid-like remnants in CKO mice and the thymus from control mice 12 weeks post induction of Meis1 deletion. Tissue sections were double stained with the indicated antibody combinations. Scale bars, 100 µm. (TIF) [file pone.0089885.s005.tif]

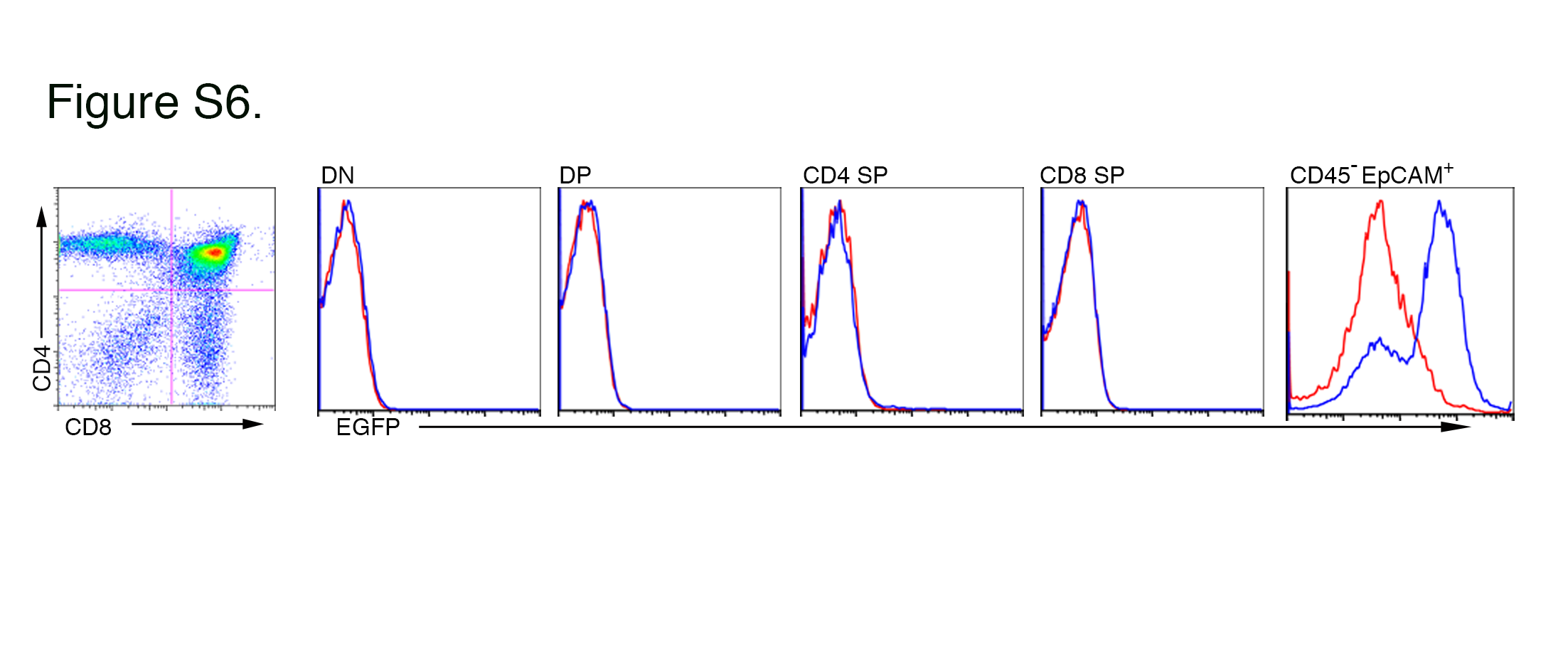

Supplement: Figure S6 — Flow cytometric analysis of Meis1 expression in the thymus by using Meis1-EGFP reporter mice. (TIF) [file pone.0089885.s006.tif]
